# Balancing Control and Complexity in Field Studies of Neonicotinoids and Honey Bee Health

**DOI:** 10.3390/insects4010153

**Published:** 2013-03-05

**Authors:** Sainath Suryanarayanan

**Affiliations:** Department of Community & Environmental Sociology, University of Wisconsin-Madison, Madison, WI 53705, USA; E-Mail: suryanas@entomology.wisc.edu; Tel.: +1-608-698-0592

**Keywords:** bee, field study, insecticide, colony collapse disorder

## Abstract

Amidst ongoing declines in honey bee health, the contributory role of the newer systemic insecticides continues to be intensely debated. Scores of toxicological field experiments, which bee scientists and regulators in the United States have looked to for definitive causal evidence, indicate a lack of support. This paper analyzes the methodological norms that shape the design and interpretation of field toxicological studies. I argue that contemporary field studies of honey bees and pesticides are underpinned by a “control-oriented” approach, which precludes a serious investigation of the indirect and multifactorial ways in which pesticides could drive declines in honey bee health. I trace the historical rise to prominence of this approach in honey bee toxicology to the development of entomology as a science of insecticide development in the United States. Drawing on “complexity-oriented” knowledge practices in ecology, epidemiology, beekeeping and sociology, I suggest an alternative socio-ecological systems approach, which would entail *in situ* studies that are less concerned with isolating individual factors and more attentive to the interactive and place-based mix of factors affecting honey bee health.

## 1. Introduction

The viability of multiple agricultural crops and broader ecosystems is threatened by unsustainable declines in the populations of honey bees and other insect pollinators in North America and elsewhere [1,2,3]. Since 2007, public attention in the U.S. and world-wide has been riveted by reports of drastic drops in honey bees, fueled by a phenomenon that came to be called Colony Collapse Disorder or CCD [4,5]. Beginning in the winter of 2004–2005, many beekeeping operations across the U.S., especially those involved in crop pollination, experienced unprecedented losses between 30–90% of their managed honey bee hives. Managed honey bees disappeared from their colonies, leaving behind the queen, young bees and large stores of honey and pollen. More than half a decade since beekeepers first saw their bees vanish, annual hive losses have remained troublingly high, and frequently over 30% [6,7]. Scientific investigations of CCD suggest that microbial pathogens are causally involved [5,8,9,10]. However, the fact that different scientific studies have identified different sets of associated microbial pathogens has led researchers to suggest that the discovered pathogens are secondary infections, with the primary causes yet-to-be-identified [3,5,6]. The current consensus among scientists is that accelerated hive losses are being caused by a combination of multiple factors, including certain agricultural pesticides, beekeeper-applied chemicals, poor nutrition, microbial pathogens and parasites [6]. But which of these factors or sets of factors play a more prominent role in causing CCD remains uncertain, with the influence of some newer kinds of agricultural insecticides being a major source of controversy.

Commercial beekeepers first discovered CCD during the course of crop pollination operations, when they traveled around the U.S. renting out their hives to farmers [4]. While there are serious disagreements within the highly heterogeneous U.S. beekeeping community about CCD and its causes, several long-time beekeepers have consistently claimed a correlation between the occurrence of CCD symptoms and the proximity of hives to agricultural crops that were treated with relatively new classes of “systemic” agricultural insecticides [11]. Labeled by the U.S. Environmental Protection Agency (EPA) as “reduced-risk”, newer systemic insecticides, such as the “neonicotinoids”—imidacloprid and clothianidin—persist for extended periods in treated plants, and accumulate in plant pollen and nectar [12]. *In-situ* beekeeper-initiated field studies suggest that CCD is *primarily* due to the *progressive* and *interactive* effects on developing bees of chronic exposure to “sub-lethal” levels of these insecticides, which are brought back to the hive by bees foraging on contaminated pollen and nectar [11]. However, scores of *toxicological* field studies show a lack of conclusive evidence in support of a prominent role for the neonicotinoids in ongoing declines of honey bees ([13,14,15], but see [16]). Also several scientists, regulatory agency officials, agroindustry representatives, and “scientific” beekeepers (beekeepers with a self-proclaimed “scientific” bent toward beekeeping) privilege these experiments as more realistic and objective assessments. In a thorough review in the journal *Ecotoxicology*, Blacquière *et al*. [17] summed up the scientific consensus by stating “[m]any lethal and sublethal effects of neonicotinoid insecticides on bees have been described in laboratory studies, however, *no effects were observed in field studies with field-realistic dosages*” (p. 988, my emphasis) [18]. In a similar vein, writing in the prestigious international journal *Science*, Ratnieks and Carreck [19] pointed to toxicological field studies by agrochemical industry and academic scientists to assert that “[a]fter 10 years of research, it seems unlikely that imidacloprid was responsible for … bee deaths.” (p. 153). Based on such “field pollinator studies” and “toxicity tests”, the EPA, which is the premier federal agency for pesticide regulation, has dismissed calls for the suspension of neonicotinoids in relation to incidents of honey bee die-offs, by asserting, for example, that “there is currently no evidence *directly* linking pesticides, including the neonicotinoid insecticides, to CCD or other serious impacts to colonies …” [20] (my emphasis).

In this paper, I examine how norms of appropriate research methods, standards of evidence, and grounds for conclusions in the environmental toxicology of honey bees become (historically) established to shape what scientists, regulators and various stakeholders know and do not know about the role of neonicotinoids in the decline of honey bee populations. I will begin with an analysis of two representative field experiments by Cutler and Scott-Dupree [14] and Dively *et al*. [15], and the key qualities that characterize the experimental approach taken in them by academic, regulatory, and industrial scientists. I will argue that the prevalent form of research regarding links between neonicotinoids and honey bee health in “the field” is the result of methodological *choices* that do not necessarily reflect “nature” or the ground realities of commercial pollination, but rather the intertwined social and historical development of professional entomology and honey bee science in the U.S. I will suggest that there is nothing objectively superior about the prevalent experimental approach to field studies of honey bees and neonicotinoids that scientists choose to accept. I will then lay out a framework for alternative approaches to field studies that draw upon conceptual and methodological perspectives from epidemiology, ecology and commercial beekeeping, and conclude by discussing the implications of adopting these for our understanding of pesticides and honey bee health.

## 2. “Control-Oriented” Field Studies

In laboratory studies, honey bee scientists (mostly in Europe) exposed individual honey bees to field-realistic dosages of the newer systemic insecticides. In the process they documented “sub-lethal” and “chronic” effects on honey bee learning, behavior, and development [17,21]. Their lab studies further suggest enhancement of the toxicity of some of these insecticides to honey bees through synergistic interactions with other synthetic toxins and pathogens [22,23]. However, because highly controlled laboratory experiments preclude consideration of other factors, such as the social organization of honey bees in whole hives, any laboratory evidence of a major role for these chemicals in declining bee health is considered to be at best *suggestive* by academic scientists, EPA officials and agroindustry representatives [24,25,26].

Regulators and university bee scientists in the U.S. have looked to field experiments as a *more definitive* source of causal evidence of the link between newer systemic insecticides and the ongoing honey bee declines. In a recent review of “pesticides and honey bee toxicity”, university bee scientists Reed Johnson, Marion Ellis and Maryanne Frazier wrote: “[a]cute toxicity tests on adult honey bees may be particularly ill-suited for the testing of systemic pesticides because of the different route of exposure bees are likely to experience in field applications. Chronic feeding tests *using whole colonies may provide a better way* to quantify the effects of systemics” [25] (p. 3, my emphasis). Such studies typically involve statistically based comparisons between a set of experimentally “treated” hives, which are exposed to a range of insecticidal doses, and a set of “untreated” control hives, while other environmental variables such as levels of nutrition, temperature and location are controlled. Chronic feeding studies in the field, by industry, university, and federal scientists, where honey bee colonies were exposed to field-relevant levels of the neonicotinoids have found very little evidence of harm, let alone any link to CCD. Below, I look at two representative field studies.

In 2007, bee scientist Cynthia Scott-Dupree’s group at the University of Guelph in Canada published a study [14] in the *Journal of Economic Entomology*, concluding that “exposure to clothianidin seed-treated Canola has no long-term impact on honey bees” (p. 765). This field experiment was designed so as to expose honey bee colonies for three continuous weeks to newly planted plots of Canola, whose seeds had been treated with or without maximally permitted levels of the neonicotinoid, clothianidin. Following the three weeks of exposure, colonies were moved to a distant yard, where they were monitored for several more months. During the course of the study, Scott-Dupree’s group assessed various measures of hive health, including the amount of honey produced, the amount of brood, and the longevity of the worker bees. They did not find any statistically significant differences between the treated and untreated colonies.

In 2008, insect toxicologist Galen Dively’s group at the University of Maryland in collaboration with bee scientists Jeff Pettis, Jody Johnson and Dennis vanEngelsdorp performed a chronic toxicity field study that was sponsored by the EPA [15]. They exposed honey bee colonies to Admire^®^, a commercial imidacloprid formulation, over a period of nine consecutive weeks. The insecticide was administered in “pollen patties” that were spiked with increasingly higher dosages, including field-relevant levels. Again, at low exposure levels the scientists saw relatively little effect of the insecticide on various measures of hive health, such as the number of adult bees, the amount of brood, the amount of food stored by bees in the hives, and the rate at which hives replaced lost queens with new ones [15]. It is worth noting that in a subsequent laboratory study, when newly emerged (one-day old) adult bees from the very same field study colonies were experimentally inoculated with *Nosema*, a common gut pathogen, bees from the field-relevant dosage group were significantly more vulnerable to infection than bees from untreated hives [27]. However, EPA officials [28] and “scientific beekeepers” [29] have questioned the “biological relevancy” of this latter laboratory result to bee colonies under “*natural* conditions”, with the EPA deeming it “highly uncertain” ([28]: p. 12–13, my emphasis), since there was no observed correlation in the field experiment between colony-wide levels of *Nosema* and the varying dosages of imidacloprid that “would have been predicted by the lab study” ([27]: p.156).

Such field experiments to investigate the links between honey bee health and the newer systemic insecticides are designed to isolate *individual* factors and their *direct*, *causal* roles. Here the onus is on precise experimental control of other potentially confounding environmental variables; it is a “control-oriented” approach [30]. This focus on experimental control occurs at the cost of understanding the ecological complexity in which honey bees and beekeepers operate, leading to what Kirk and Kutchins [31] have called “the substitution of precision for validity”. In Cutler and Scott-Dupree’s [14] study, the treated and control field plots were separated by a mere 300 meters. As a result, so-called untreated colonies, while not receiving pesticides from the experimenters, could have had bees that foraged on the relatively nearby clothianidin-treated Canola. Similarly, so-called treated colonies could also have had bees that foraged on the nearby untreated plot of Canola. In other words, the observed “no long-term impact on honey bees” from clothianidin may have been because all study colonies inadvertently had access to both clothianidin-treated and untreated experimental plots. Indeed, the experimenters detected clothianidin at low levels in some hives from both the treatment and the control groups [14]. The reality is that honey bees do not just stay on one plot but can travel over six kilometers to forage [32]. In setting up the control plots so close to the treated ones, Scott-Dupree’s group overlooked this fact. Here, the reality and complexity of the experience of bees in the field is ignored, and the results may not be representative. In Dively *et al*.’s [15] field study from 2008, researchers affixed “pollen traps” on the front entrance of each honey bee colony to cut down on the amount of nutritive pollen that forager bees brought into the colony. By doing so, the researchers aimed to not only “induce” bees to ingest the experimentally provisioned diet, but also to control any variation between colonies due to differences in nutritional intake. Since bees in all the study colonies could only access a single source of artificial pollen substitute, this “control-oriented” manipulation may have had an across-the-board depressive effect on colony health; which in turn could have affected how different colonies responded to insecticidal exposure. In both field studies, the manipulations that were carried out to control specific environmental variables in fact led to the introduction of new and unanticipated complications that could have influenced the eventual results. My point is not that the experimental controls in these studies were bad, but that the focus on isolating the *direct* effects of an *individual* insecticide on honey bee colonies creates an experimental system where plausible and equally important *indirect* effects as a result of *cumulative interactions* between the chemical and the experimentally controlled factors are obscured.

In focusing on only one or two factors, contemporary field study designs tend to exclude more complex scenarios, wherein the newer systemics by themselves may not be causing honey bee die-offs, but may do so through intricate interactions with multiple other environmental factors across a honey bee’s lifecycle and over a longer-term [33]. Managed honey bees and other insect pollinators are exposed throughout their lifecycle to a multitude of local environmental factors, including nutrition, pesticides, pathogens, and parasites, many of which are known to interact with the newer systemic insecticides. For example, a recent survey of honey bee hives in North American beekeeping operations found them to contain an average of 121 different pesticides and their toxic break-down products [34]. Such pesticide residue survey results of North American commercial apiaries have found very high levels of beekeeper-applied miticides rather than grower-applied systemic insecticides in honey bee colonies [5,34,35]. That said, the authors of these surveys point out that these results by no means constitute conclusive evidence that colonies did not experience damaging exposure to systemic chemicals [5]. Nor do these surveys rule out the realistic possibility that systemic chemicals and their toxic break-down products exist in colonies at levels below instrument detection limits, where they can still have effects on developing honey bees through interactions with other prevalent pesticides and pathogens [27].

It is not that bee scientists and toxicologists are unaware of the shortcomings of a “control-oriented” approach; quite to the contrary. In a meeting with the EPA’s Pesticide Program Dialog Committee in 2008, Jeff Pettis, the lead bee scientist of the U.S. Department of Agriculture (USDA) explained that “in research, we like to eliminate all other variables and focus on one thing” because “[i]ts much easier to test for”. And as a result, Pettis believes that scientists “tend to ignore interaction” ([24]: p. 228). Although “it’s very hard to set up experiments to test interactive effects” these are beginning to be looked at “at both university and at the federal labs” ([24]: p. 229). However, cutting-edge lab studies such as Pettis *et al*. [27] are considered to be of lesser value by regulators, beekeepers and other academic and agroindustry scientists, who question their relevance for bees in the “real world,” outside of the confines of laboratories. In other words, even though federal, university, and agrochemical industry scientists conceptualize the current decline in honey bee health as a complex multifactorial phenomenon [6,26], their “control-oriented” evidentiary practices for evaluating the real-world links between pesticides and the ongoing die-offs emphasize the direct effects of individual factors. So, the purported lack of conclusive evidence in support of a prominent role for the newer systemic insecticides may in fact be due to the “control-oriented” methodological assumptions that drive prevalent field experiments.

Through analyses of semi-structured interviews with relevant stakeholders, published documents, and ethnographic research at a bee biology laboratory that is examining the links between agrochemicals and CCD, my prior research has illuminated how a particular set of “control-oriented” research norms and practices from agricultural entomology came to dominate scientific investigations of the links between pesticides and honey bees, and in the process marginalize the knowledge claims and positions of several beekeepers in the CCD controversy over the newer systemic insecticides [36]. The primacy of “control-oriented” toxicological field studies in scientific, regulatory, and industrial understandings of pesticides and honey bee health is not because they are objectively superior, but because of a particular history of U.S. agriculture and academic science. The rise to prominence of a control-oriented approach can be traced to the unfolding of entomology as a science of insecticide development in the U.S. Entomology developed as a scientific discipline at the turn of the 20th-century in the context of *agricultural research* in the U.S. Early entomologists at land grant universities, state agricultural experimental stations, and at the USDA attempted to enhance their professional positions by convincing growers and others that extant problems in agriculture were mainly due to insect pests, and that they possessed the appropriate knowhow to provide the solutions [37,38,39]. State entomologists experimented predominantly with various kinds of chemical control, and demonstrated their success through a causal approach, which highlighted relatively rapid, and easily quantifiable lethal effects of individual chemicals on “target” insects [39]. The modus operandi of early honey bee scientists to insecticides and “non-target” bees also came to adopt this approach [40,41,42], reflecting the influence of the norms of the agricultural organizations for which they worked, including the USDA, and their professional stakes in the overlapping research communities where they sought peer recognition.

Early bee scientists doused “non-target” bees in laboratory and field settings with pre-determined amounts of individual chemicals and measured various indices of mortality relative to “control” (untreated) bees [40]. In the lab, scientists traced “dosage mortality” curves to calculate the LD_50_—the dose of a chemical at which 50 percent of the exposed group of bees is killed within a period of 2–3 days—and used it to rank the relative lethal toxicity of “organic insecticides” such as gamma benzene hexachloride and DDT to honey bees [42]. In the field, scientists set up miniaturized honey bee colonies (“nucs”) confined in screened cages over crops that had been subjected to varying doses and timings of different pesticide applications [41]. Following different durations of exposure, they documented shifts in percent mortality, numbers of brood and amounts of stored nectar and pollen among the caged honey bees [41,42]. Bee scientists conveyed the results of their experimental investigations to beekeepers and growers in trade journals and extension periodicals [40], and to their entomologist peers in premier scientific journals such as the *Journal of Economic Entomology* [42].

These investigations of pesticides in relation to “non-target” honey bees by bee scientists were underpinned by the very same “control-oriented” experimental approach that characterized their peer entomologists’ efforts to help develop more effectively killing chemicals against “target” insects. Similar to the latter, bee scientists emphasized the relatively rapid, lethal effects of individual pesticides on honey bee health in their studies. This then meant that *sub-lethal* effects of pesticides in *interaction* with shifts in other ambient factors such as fragmentation of the landscape, nutritional availability, quality and diversity, pathogens, and exposure to other synthetic chemicals occurring *over multiple generations* of a bee hive’s lifecycle were not given equivalent consideration by these scientists. Thus, based predominantly on such a “control-oriented’ approach, Anderson and Atkins [41], who were extension and research scientists at the University of California, concluded in 1968 that in general “modern pesticides … are less hazardous to honey bees” and “although the newer pesticides are used in greater quantities over larger areas and over a greater variety of crops … they can usually be used with safety if the … facts [from studies] and precautions are taken into consideration” (p. 231) (quoted in [36]).

In the aftermath of widespread public concerns about the negative effects of industrialized chemicals on human health and the environment in the 1960s, regulatory and academic interest in the real world effects of pesticides on “bioindicators” such as honey bees substantially increased. Since then, the prevalent “control-oriented” approach has come to influence and be further shaped by academic bee scientists, for whom the issue is first and foremost a *research* problem that is geared toward the norms of professional success in peer institutions, such as in university departments, scholarly academic journals and grant funding agencies. The high stakes involved in securing publications, grants and tenure, orient academic bee scientists toward adopting historically established control-oriented research norms and practices, because they have a higher likelihood of leading to conclusive and publishable results. Furthermore, whether results from field experiments are “conclusive” is based on a statistical cut-off at a 95% confidence interval; this criterion is a social convention. It glosses over the enormous variability that is present in field settings both between and within honey bee colonies. As a result, experimental field studies tend toward concluding “no differences” between pesticide-treated and untreated honey bee colonies, when in fact there might be a difference. This preference for false negative results (Type II error), where a substance is incorrectly concluded to be safe when in fact it is harmful, is compatible with the livelihood stakes of academic scientists. This is because a false negative would lead a researcher to miss a discovery but not damage his or her reputation to the same degree as a false positive, which might be more professionally damaging when it is revealed to be incorrect and causes the researcher to retract the publication [43].

The *gaps* created by the control-oriented character of field research pertaining to pesticides and honey bee health are further maintained by the career structure of academia. Field research about the cumulative effects of the newer systemic insecticides in interaction with other factors remains largely “undone”, in part because it is very hard to measure [33,44]. As a result, there is little incentive to do serious research on hard-to-measure issues such as those involving low level interactions between pesticides and other environmental factors, where there is a high risk of getting inconclusive evidence, since this would hurt the prospects of getting grants, publications and tenure. To be clear—I am not suggesting here that certain (groups of) entomologists or honey bee scientists self-consciously or strategically manipulated knowledge practices to suit their own ends. My main argument is that state, academic and industry entomologists and honey bee scientists are bound together in a *shared culture* of formal and informal norms, taken-for-granted assumptions, and research practices, which lead these scientists to choose certain research agendas, methods and modes of interpretations. The limits of existing field experiments on pesticides and honey bees point to the need for a revised culture of conceptual frameworks, research norms, and modes of knowledge production that emphasizes a better balance between experimental control and environmental complexity.

## 3. “Complexity-Oriented” Field Studies

A “complexity-oriented” approach to research on issues of honey bee health would entail “an openness toward unanticipated events as well as uncontrollable and *context-sensitive* settings” ([30]: 790, my emphasis). “Context” here refers not only to the geographical and temporal dimensions of the settings in which honey bees operate, but also to the human (social and historical) dimensions. Complexity-oriented approaches need not be fundamentally at odds with control-oriented approaches; they would still need to incorporate some measure of control, since too much openness to complexity would drown any “signal” of relevance in a flood of “noise”, rendering the results literally incomprehensible. The key issue here is not whether to control, but what to control, how, and towards what end. One way to introduce complexity, while also addressing some of the imperatives of control prevalent in entomology, would be to involve scientists from a broader array of disciplines, whose experimental approaches to control consider the dynamics of complex adaptive systems. In other words, balancing imperatives of control and complexity in field studies depends crucially on who the involved actors are, and what their professional and/or disciplinary orientations are. I argued earlier on that the prevalent “control-oriented” approach to the design and interpretation of field experiments regarding pesticides and honey bees reflects the disciplinary commitments and values of a narrow range of scientists, and as a result, that toxicological field experiments remain partial in their scope to address the multifactorial and complex “ground realities” that managed honey bees and beekeepers face in fuller and fairer ways. This predicament points to the need for a meaningful inclusion of more “complexity-oriented” modes of knowledge production and practitioners from (1) scientific fields such as ecology and epidemiology, which routinely analyze complex, multifactorial phenomena such as lake ecosystems [45] and human populations [46], (2) “on-the-ground” stakeholder communities such as those of beekeepers”, whose “feel for the organism” approach is equally crucial for achieving a deeper understanding of bee health [13], and from (3) humanistic fields that analyze the interplay between honey bee colonies and human institutions, policies and practices [36,44,47,48].

In this regard, the emergence of descriptive field studies of honey bee health [5] and the “Bee Informed Partnership” (BIP) [49] are welcome developments. The BIP initiative seeks to identify “risk factors” of honey bee disease *and* beekeeping management practices “to reduce risk-factor exposure” by drawing explicitly on an epidemiological approach [49]. Honey bee epidemiologists investigate patterns of disease occurrence and their causal factors in honey bee populations. For example, vanEngelsdorp *et al*. [5] performed an “epizootiological” analysis of hive samples from CCD and “non-CCD” apiaries in order to identify “clues” to the cause(s) of CCD. Based on differences in the levels of various measured “risk factors”, such as pathogen loads and pesticide residues at an alpha-level of 95%, the scientists concluded whether any individual risk factor was likely/not likely to be associated with CCD. In providing a thick description of potentially influential factors in CCD and non-CCD colonies, studies such as vanEngelsdorp *et al*. [5] and the BIP initiative [49] have several valuable elements of what I take to be complexity-oriented field research. However, to the extent that the influence of each risk factor is analyzed individually, knowledge gaps will remain regarding the combinatorial effects of multiple factors. Furthermore, the conditions under which any particular risk factor is recognized as being of significant concern continue to be based on the traditional academic convention of 95% statistical certainty. This therefore exemplifies a preference for type II errors, that is, a false negative orientation, wherein a risk factor is more likely to be considered not involved, when in fact it is. By contrast, primarily affected commercial beekeepers might prefer scientists to “err on the side of caution” and make a type I error (a false positive), rather than a type II error [11].

Ecologists are likely to look at the issue of pesticides and honey bee health from a “systems” perspective, where the analytic focus would be on patterns and processes of interaction between different components across varying levels of organization, scale and time. Control-oriented notions of experimental control, replication, and statistical interpretation become problematic when faced with the dynamics of a complex system such as an entire lake (an “n” of 1), where it is simply not feasible to ask the kinds of narrow cause-effect questions or to carry out the sorts of replications that a control-oriented approach would entail. This scenario is arguably true for honey bees as well. Each honey bee colony is conceivably a “complex adaptive system”; a “superorganism” [50], which can issue differential compensatory responses to the same stimulus (e.g., pesticide exposure) under shifting contexts such as those faced by migratory beekeeping operations, thus complicating any control-oriented attempt at prediction, replication, and generalization. An ecological systems approach would lend itself to the analysis of an enduring conundrum in our understanding of a honey bee colony as a superorganism: what are the colony-level dynamics that allow for a colony to buffer and survive through periods of cumulative exposure to environmental stressors, such as to sub-lethal levels of neonicotinyl insecticides [50]?

Moreover, while it is essential to ask what “risk factors” diseased and healthy colonies are exposed to, this is not at all sufficient. Complexity-oriented field research on honey bee health would prompt scientists to ask *why* some colonies but not others are exposed to certain risk factors, and what other kinds of changes occur that lead to the exposures. In other words, even if honey bee epidemiologists identified certain pesticides to be prominently associated with a particular bee disease, the *social* circumstances that *mediate* this exposure remain to be fully integrated as a crucial aspect of the broader analysis. Local human activities, including science, beekeeping, and agricultural policies and practices, play an integral role in honey bee colony health, and this calls for an approach that conceptualizes honey bee health in terms of a *socio-ecological systems* framework [51]. While vanEngelsdorp *et al*.’s BIP initiative [49] recognizes the importance of context to beekeeping and honey bee health, their view of context is relegated to an *economic* one. There is no doubt that the costs of managing honey bee colonies constitute a significant “bottom-line” concern for commercial beekeepers. But that bottom-line is in itself a consequence of social factors, such as government policies and yield-oriented institutional logics and practices, which led to huge increases since World War II in monocropping operations, pesticide and fertilizer usages, and accompanying die-offs of endemic pollinators [52,53,54].

The socio-ecological systems framework emerged during the last decades of the 20th-century amidst an increasing realization that ecological research must incorporate human impacts, and conversely, sociological research must incorporate ecological effects. Through a series of case studies, Liu *et al*. [55] outlined how an integrated approach to “coupled human and natural systems” shows “new and complex patterns and processes not evident when studied by social or natural scientists separately” ([55], p. 1513). In examining complex reciprocal interactions and feedback loops between human and natural systems, this approach measures ecological variables such as landscape effects, as well as social factors such as government policies, across multiple spatio-temporal scales.

A socio-ecological systems approach to field studies of honey bee health would seek to shed light on how multiple factors across social, historical and ecological contexts interact to influence ongoing patterns of honey bee health. It would involve humanistic researchers versed in analyzing the complicated interplay between cultural norms, governmental policies, and fact and artifacts in scientific and non-scientific fields [36,44,47,48].

The design and implementation of a viable socio-ecological system approach to field studies of pesticides and honey bee health would also need to involve beekeepers and their field knowledge in a meaningful and egalitarian manner. Informal beekeeping practices of gauging hive health and disease constitute a highly valuable source of “local” knowledge about the dynamic and multidimensional aspects of honey bee colonies. For example, beekeepers assess brood health by identifying the overall pattern in which various developmental stages of honey bee brood are distributed across a colony’s comb. The “brood pattern” additionally provides information about the queen’s health, local nutritional resources, and the presence of diseases. Informal measures, such as brood pattern, produce knowledge that is unquestionably practical, which means that commercial beekeepers will seriously consider the influence of multiple environmental factors, not just those that are easily isolatable and definitive from the standpoint of control-oriented scientific practices. Of course, commercial beekeepers’ approach to understanding the factors that affect bee health is shaped by their stakes in keeping colonies healthy and alive. It engenders knowledge that errs on the side of arriving at false positive conclusions (Type I error), a precautionary approach where a substance that is safe might be incorrectly labeled as harmful.

While it is all well and good to call for greater beekeeper-scientist collaborations in the design, implementation and interpretation of field studies, the reality is that in the eyes of many academic, regulatory and agro-industry scientists, beekeepers’ knowledge of bee health is “anecdotal”, “simply trial and error”, and “not data” [36]. So, simply bringing beekeepers across the table with scientists will not be enough to achieve a genuinely collaborative field study, because of factors such as trust and asymmetries in power and resources between these heterogeneous groups. Here, humanists with the background to design and facilitate egalitarian trust-building interactions between beekeepers, bee scientists, and other stakeholders would be a crucial step toward the development of a robust socio-ecological systems approach to field studies of honey bees and pesticides.

In other words, what I am calling for is the development of a *transdisciplinary* set of “theoretical structures, research methods and modes of practice” [56] that extend beyond academic disciplinary silos to incorporate approaches rooted in other intellectual fields, such as ecosystem ecology and the interpretive social sciences, as well as involve non-scientist and non-academic professional fields, such as those of commercial beekeepers. The field research would likely involve qualitative as well as quantitative measures, be less precisely controlled, more correlative, than the standard research, and give preference to false positive over false negative errors. This is liable to produce only suggestive results of limited generalizability from the standpoint of academic entomology and toxicology. At the same time, the field experiments would be of direct utility to beekeepers, farmers, university extension agents, and crop consultants, since I anticipate that it would lead to the development of a diagnostic list of mixes of different factors and how they impinge upon honey bee health in particular settings.

How might such transdisciplinary collaborative research take shape around examining the purported links between newer systemic insecticides and honey bee health? At the outset, it would entail a series of structured deliberations over the course of two years between key stakeholders. Deliberants with diverse methodological and conceptual tools for place-based analyses, high levels of credibility among professional colleagues, a history of productive relationships, and an openness to non-traditional approaches, would be recruited from an array of relevant stakeholder groups—beekeepers, growers, university scientists (pollination biologist, ecosystem ecologist, social insect biologist), university sociologists, extension agents, and public officials. Facilitated deliberations between participants would be interlaced with a pilot field study on honey bees, which would be designed, implemented and refined based on the ongoing deliberations. Subject to revision by the deliberants, the pilot field trial would span two successive summers, and would compare the relative health of honey bee colonies across four field-sites: monocropped fields and polycropped fields managed with or without “reduced risk” systemic insecticides. Deliberants would learn about each other’s knowledge and practices, discuss the design features and measures, and engage with broader stakeholder communities, in order to arrive at an agreed upon experimental design, which would subsequently get implemented.

## 4. Conclusions

The main aim of this paper is to prompt bee scientists to consider revising the existing norms and practices of experimental field research regarding pesticides and honey bees so as to better incorporate the social and ecological complexity in which honey bees and beekeepers operate. The worlds of scientist and beekeeper research about large-scale declines in honey bee populations, including CCD, have been largely separate, with beekeepers’ field knowledge pointing to a prominent role for a new set of insecticides, and toxicological field experiments pointing to a lack of conclusive evidence supporting beekeepers’ claims. Based on my ethnographic and socio-historical research [36,44], I argued here that a set of “control-oriented” toxicological research norms and practices, which emphasize the isolation of individual causal factors and their direct effects, have come to dominate field investigations of the links between pesticides and honey bee health. As a result, serious analyses of the cumulative and interactive effects of pesticides in combination with other ambient environmental factors—a dynamic that beekeepers’ place-based analyses point to—tend to be precluded, and their knowledge claims and policy positions are marginalized in debates over the cause(s) of the die-offs. We need a fuller set of methodologies that balance and combine experimental control with more “complexity-oriented” modes of analysis. In this regard, I discussed the various elements of an alternative socio-ecological systems approach to honey bee field research, one that would be sensitive to the multiple environmental and social contexts in which honey bees exist. The envisioned research endeavor would entail equitably structured collaborations between a broader array of knowledge producers—including from honey bee biology, ecology, commercial beekeeping, and the sociology and anthropology of science, whose everyday practices grapple with place-based social and ecological complexities—by involving them in facilitated discussions with other relevant scientists and policymakers toward designing field research. Since the problem of how pesticides and other multiple factors interact with honey bee health in the real world is a matter of scientific as well as public concern that is marked by high levels of complexity and uncertainty, its successful resolution requires a transdisciplinary approach to experimental field research of the sort that I laid out in this paper. Field studies based on this alternative approach stand to provide concerned farmers, beekeepers and other stakeholders with *practical* information. Findings would also address current gaps in the scientific realms regarding the mechanisms that mediate the interactive effects and buffering capacities of honey bee colonies.
